# Galectins-1, -3 and -9 in leukemia: mechanistic insights and therapeutic translation

**DOI:** 10.3389/fimmu.2025.1685266

**Published:** 2025-09-25

**Authors:** Tianning Wang, Yanxin Zhang, Yuchuan Guo, Mengmeng Zhao, Xiaojun Cai, Shaojie Feng

**Affiliations:** ^1^ Research Center of Translational Medicine, Central Hospital Affiliated to Shandong First Medical University, Jinan, China; ^2^ College of Chemical Engineering, Department of Pharmaceutical Engineering, Northwest University, Xi’an, China; ^3^ Shandong Junteng Medical Technology Co., LTD, Jinan, China; ^4^ Department of Cardiology, The Second Hospital of Shandong University, Jinan, China

**Keywords:** galectin, leukemia, diagnosis, molecular mechanism, inhibitors

## Abstract

Galectins, β-galactoside-binding proteins, function as key regulators in pathological transitions, bridging tissue homeostasis to oncogenesis and inflammation through intracellular and extracellular mechanisms. Notably, they play a pivotal role in the pathogenesis of leukemia by interacting with glycoconjugates to promote tumor progression. Among them, Galectin-1 (Gal-1), Gal-3, and Gal-9 have been associated with multiple leukemia subtypes, such as acute myeloid leukemia (AML), acute promyelocytic leukemia (APL), B-cell precursor acute lymphoblastic leukemia (BCP-ALL), adult T-cell leukemia (ATL), and chronic lymphocytic leukemia (CLL). These galectins contribute to leukemic cell survival by modulating extracellular matrix (ECM) interactions, suppressing anti-tumor immune responses, and promoting immune escape. Their involvement in sustaining leukemic proliferation and immune evasion highlights their potential as therapeutic targets. Recent advancements in the development of galectin inhibitors provide promising avenues to disrupt these oncogenic pathways. However, distinct galectin isoform pathologies across diseases require highly selective therapeutics, and substantial carbohydrate recognition domain (CRD) structural homology combined with conserved β-D-galactopyranoside-binding mechanisms complicates specific inhibitor design. This review summarizes galectin-mediated mechanisms in leukemia biology, evaluates the potential of galectin-targeted therapies and offers insights for the development of specific inhibitors of Gal-1, -3, and -9 to promote clinical management and treatment efficacy.

## 1 Introduction

**Figure 1:**
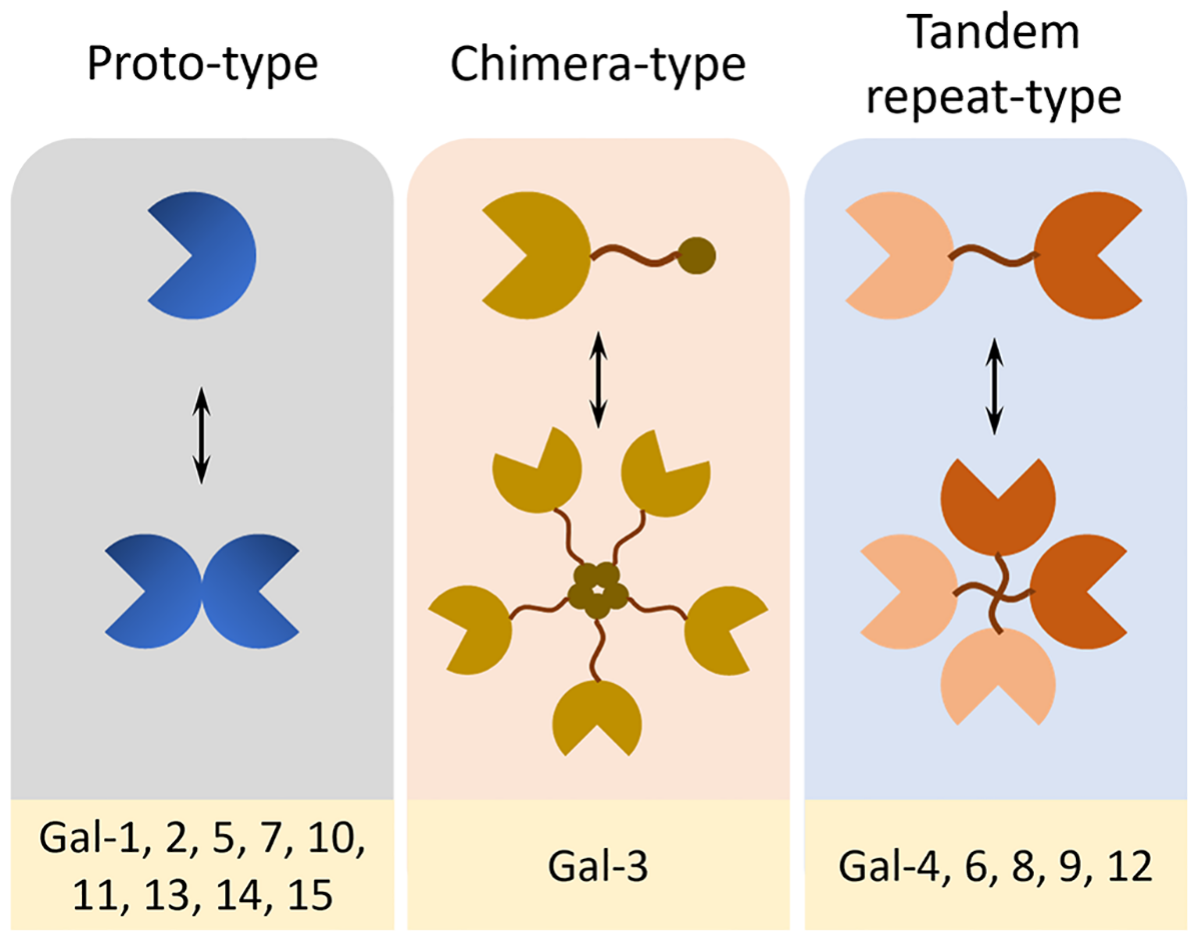
Structural classification of galectins. Galectins are classified into three structural subtypes based on their domain architecture and oligomerization patterns. Proto-type galectins (Gal-1, -2, -5, -7, -10, -11, -13, -14, and -15) contain a single CRD and exist either as monomers or non-covalent homodimers. Chimera-type galectin (Gal-3) is a single CRD related to an N-terminal proline-rich domain that allows oligomerization *via* non-lectin interactions. Tandem-repeat type galectins (Gal-4, -6, -8, -9, and -12) possess two distinct CRDs connected by a flexible polypeptide linker, allowing for bivalent glycan binding and functional diversity.

Galectins (Gal) are a family of soluble lectins with a conserved affinity for β-galactoside-containing glycans (1). Structurally, they are classified into three subtypes: proto-type galectins (Gal-1, -2, -5, -7, -10, -11, -13, -14, -15), which function as monomers or noncovalent homodimers containing identical carbohydrate recognition domains (CRDs); tandem-repeat type galectins (Gal-4, -6, -8, -9, -12), characterized by two distinct CRDs connected *via* a flexible linker; and chimera-type galectins (Gal-3), comprised a single CRD, an intermediary proline–glycine–alanine–tyrosine repeat domain, and a short N-terminal domain that mediates oligomerization (**Figure 1**) (2). Functionally, galectins are key regulators of pathological transitions, bridging tissue homeostasis to oncogenesis and inflammation through intracellular and extracellular mechanisms (3, 4). They contribute to the modulation of core cancer hallmarks by promoting tumor progression, immune escape, and resistance to various therapeutic modalities, including immunotherapy, chemotherapy, radiotherapy, and targeted treatments (4, 5). They also modulate fibrotic responses and inflammatory resolution by engaging in cell-type-specific signaling within the tissue microenvironment (2, 6). Acting as molecular rheostats, galectins orchestrate self-reinforcing feedback loops that sustain disease progression while simultaneously regulating the balance between pathological disruption and tissue repair *via* spatiotemporal control of intercellular communication networks (3, 7, 8).

Galectins exert multifaceted roles in leukemia development, disease progression, and therapeutic resistance in hematological malignancies. They function as prognostic biomarkers and therapeutic targets by modulating oncogenic signaling pathways, supporting leukemia stem cell (LSC) self-renewal, and facilitating metabolic reprogramming to sustain malignant proliferation (9–11). Galectins are also involved in immune evasion by inhibiting antitumor T-cell responses and enhancing the immunosuppressive activity of myeloid-derived suppressor cells (MDSCs) (11). Their role in drug resistance is further underscored by their ability to upregulate survival-related proteins such as MCL-1 and MDR-1 and to induce epigenetic modifications, which contribute to relapse and treatment-refractory disease (11). By mediating crosstalk between leukemic cells and the tumor microenvironment (TME), galectins integrate immune suppression, stemness maintenance, and adaptive survival mechanisms, thus exerting a systemic influence on leukemia biology and treatment response (9). Most of the studies in this domain have focused on Gal-1, -3, and -9. This review highlights their mechanistic roles in the initiation, progression, and drug resistance of various leukemia subtypes and discusses the development of galectin-targeted inhibitors/antagonists under investigation for potential clinical application.

## 2 Role of Gal-1 in leukemia

### 2.1 Gal-1 as a multifaceted biomarker in leukemia

Gal-1 is a biomarker of significant clinical relevance in the pathogenesis, progression, and prognosis of leukemia and related myeloid malignancies. Elevated expression of Gal-1 has been consistently associated with more aggressive disease phenotypes and poorer clinical outcomes in various leukemia subtypes. In acute myeloid leukemia (AML), high Gal-1 levels are associated with shorter disease-free survival, increased blast counts in the bone marrow (BM), and enrichment in LSCs, all of which are associated with poor overall survival (OS) and event-free survival (EFS) (12–15). Similarly, in B-cell acute lymphoblastic leukemia (B-ALL), Gal-1 is a highly sensitive and specific marker for MLL-rearranged subtypes, which are characterized by unfavorable prognoses (16). In chronic lymphocytic leukemia (CLL), elevated Gal-1 levels in both BM and plasma distinguish progressive from stable disease (17). Furthermore, Gal-1 overexpression is involved in the pathogenesis of myeloproliferative neoplasms (MPNs), where it may contribute to disease progression and transformation into secondary leukemia (18). Its prognostic relevance also comprises lymphoid cancers such as classic Hodgkin lymphoma (cHL), where higher serum and TME levels of Gal-1 are associated with high tumor burden, poor survival outcomes, and disease progression (19, 20). These results support Gal-1 as a reliable biomarker for risk stratification, therapeutic intervention, and disease monitoring in leukemia and related hematopoietic malignancies.

### 2.2 Molecular mechanisms underlying Gal-1-driven leukemogenesis and progression

#### 2.2.1 Pathogenic mechanisms

**Figure 2:**
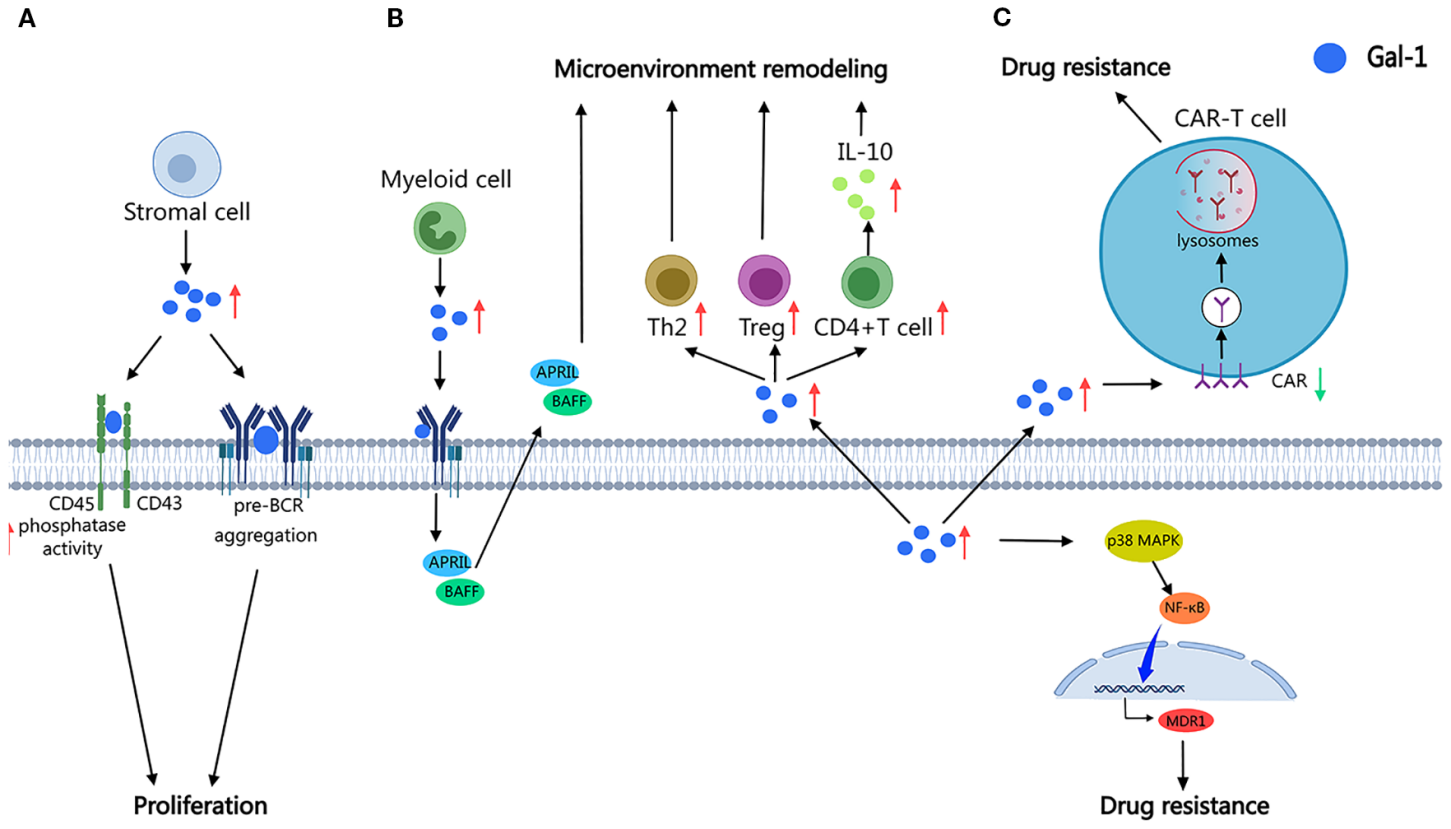
Schematic illustration of the role of Gal-1 in leukemia. Gal-1 promotes leukemia progression **(A)**, remodels the immunosuppressive microenvironment **(B)**, and induces resistance to therapeutic drugs **(C)** in various leukemia subtypes through diverse mechanisms, such as pre-BCR clustering, modulation of CD45 phosphatase activity, Th2/Treg polarization, and IL-10 induction, as well as upregulation of MDR1 and consequent drug resistance or suppression of CAR T-cells, thereby highlighting its potential as a crucial therapeutic target. (The diagram was created using MedPeer).

Gal-1 plays distinct roles in diverse leukemia subtypes through context-dependent mechanisms (**Figure 2A**). In B-cell precursor acute lymphoblastic leukemia (BCP-ALL), Gal-1 interacts with the λ5-UR domain of the pre-B cell receptor (pre-BCR), promoting the formation of large, immobile aggregates that accelerate pro-survival signaling pathways (21, 22). Similarly, in B-ALL, Gal-1, derived from the bone marrow microenvironment, promotes leukemic progression by inducing pre-BCR clustering, activating downstream signaling cascades, and promoting pre-B cell proliferation (23). In AML, Gal-1 contributes to LSC maintenance by reprogramming lipid metabolism, modulating the immunosuppressive microenvironment, enhancing cellular proliferation, and inhibiting apoptosis (13). In CLL, Gal-1 functions as a molecular bridge between CD43 and CD45, forming a ternary complex that regulates CD45 phosphatase activity, thus driving the unchecked proliferation of malignant B cells (24). These findings underscore Gal-1 as a multifaceted regulator in leukemia subtypes through signaling modulation.

#### 2.2.2 Microenvironment remodeling

Gal-1 is crucial in shaping immunosuppressive microenvironments that support leukemic progression across various malignancies (**Figure 2B**). In CLL, Gal-1 secreted by myeloid cells, such as nurse-like cells, macrophages, and dendritic cells (DCs), enhances leukemic cell activity by modulating B-cell receptor signaling or regulating BAFF/APRIL secretion, and helps to establish the appropriate microenvironmental conditions for leukemic progression (17). Moreover, elevated Gal-1 expression promotes the induction of IL-10-producing CD4^+^ T cells and drives phenotypic modulation of dendritic cells, thus establishing an immunosuppressive microenvironment that favors leukemic cell persistence (25). Similarly, in Hodgkin lymphoma, Gal-1 secreted by Reed–Sternberg cells enhances a Th2/Treg-skewed immune microenvironment, reinforcing immunosuppression and supporting tumor immune evasion (26). In AML, Gal-1 suppresses immune surveillance by reducing activated peripheral blood mononuclear cell proliferation and increasing CD4^+^ T cell prevalence, thus promoting disease progression (27). These results highlight Gal-1 as a key mediator of microenvironmental reprogramming in leukemia, driving immune evasion and tumor survival.

#### 2.2.3 Drug resistance

Gal-1 plays a pivotal role in mediating therapy resistance in leukemia through subtype-specific mechanisms (**Figure 2C**). In CML, Gal-1 overexpression activates the p38 MAPK/NF-κB signaling cascade, resulting in the upregulation of MDR1 and contributing to chemoresistance in BCR-ABL-positive leukemic cells (28). In AML, elevated Gal-1 expression downregulates CAR, impairing CAR-T cell cytotoxicity and facilitating immune escape (29). Pharmacological inhibition of Gal-1 has been shown to enhance chemosensitivity in both primary AML cells and established cell lines, highlighting its therapeutic potential in overcoming drug resistance (14). These results position Gal-1 as a key regulator of microenvironment-mediated therapeutic evasion in leukemia.

Overall, Gal-1 emerges as a pivotal player in leukemia, functioning both as a biomarker and a molecular driver. Its roles in proliferative signaling, metabolic adaptation, immune suppression, and drug resistance highlight its potential as a therapeutic target. Future research should focus on developing selective Gal-1 inhibitors (e.g., small molecules or bispecific antibodies) and investigate their potential in combination with immunotherapeutic agents or epigenetic modulators to overcome treatment resistance and improve patient survival.

## 3 Role of Gal-3 in leukemia

### 3.1 Gal-3 as a clinical indicator in leukemia

Gal-3 has emerged as a critical biomarker and potential therapeutic target in various leukemia subtypes, with its elevated expression consistently linked to adverse clinical outcomes. In APL, elevated serum levels of Gal-3 are significantly associated with reduced OS and relapse-free survival (RFS), representing an independent adverse prognostic factor for RFS in patients receiving all-trans retinoic acid (ATRA) and arsenic trioxide (ATO)-based therapies (30). Similarly, in non-M3 AML, increased Gal-3 expression correlates with lower complete remission (CR) rates, higher incidence of primary refractory disease, and inferior OS, thus establishing Gal-3 as an independent marker of poor prognosis (31, 32). In broader AML cohorts, elevated Gal-3 levels have been related to shorter remission durations and unfavorable survival outcomes (33, 34). In diffuse large B-cell lymphoma (DLBCL), upregulation of Gal-3 has been directly associated with adverse clinical prognosis (35). These results depict Gal-3 as a multifaceted regulator of leukemogenesis, disease progression, and therapeutic resistance, highlighting its dual role as a prognostic biomarker and a candidate for targeted therapy.

### 3.2 Molecular mechanisms underlying Gal-3-driven leukemogenesis

#### 3.2.1 Leukemic pathogenesis and microenvironment crosstalk

**Figure 3:**
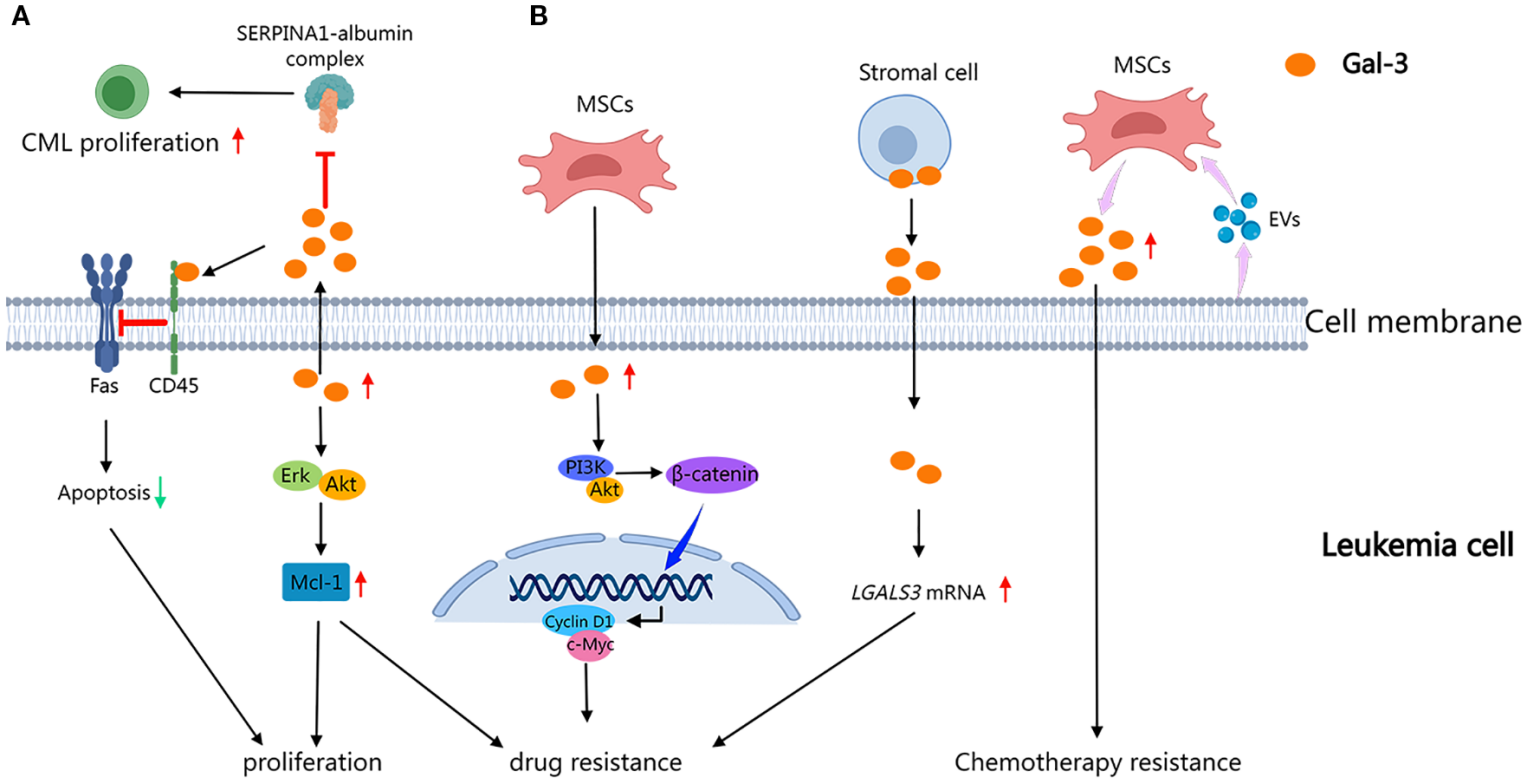
Role of Gal-3 in leukemia progression and drug resistance. Gal-3 drives leukemia progression by enhancing the survival and proliferation of leukemia cells as well as modulation of the microenvironment through key signaling pathways and stromal interactions **(A)**, while also inducing chemoresistance mediated by autocrine loops and niche remodeling **(B)**. (This diagram was created using MedPeer).

Gal-3 is multifaceted in modulating leukemia progression and microenvironmental interactions (**Figure 3A**). In AML, MSC-derived Gal-3 is critical for maintaining MSC homeostasis and regulating AML cell localization and survival within the BM niche, highlighting its importance in leukemia-stroma crosstalk (34). In acute leukemias (AL), primarily comprising AML and ALL, Gal-3 contributes to leukemic cell survival by promoting AKT-mediated inactivation of GSK3β, thus initiating anti-apoptotic, pro-proliferative, and metabolic signaling pathways (36, 37). In CML, Gal-3 overexpression induces leukemic cell proliferation, chemotaxis, and resistance to apoptosis by activating the Akt and Erk pathways and accumulating the anti-apoptotic protein Mcl-1. Gal-3 also enhances BM homing and lodgment of CML cells and bone marrow stromal cells (BMSCs), thus promoting a supportive microenvironment that drives disease progression (38). Furthermore, Gal-3 promotes paracrine growth of CML cells by disrupting the inhibitory effects of the SERPINA1-albumin complex in the TME (39). Gal-3 also contributes to apoptosis resistance by interacting with CD45, protecting B cells from anti-Fas-induced cell death in DLBCL (40, 41). Moreover, Gal-3 has been shown to interact with Mer tyrosine kinase, a mechanism that may facilitate central nervous system (CNS) relapse in ALL through feedback regulatory pathways (42). These findings revealed Gal-3 as a pivotal regulator of leukemia cell survival, proliferation, and microenvironmental adaptation, positioning it as a potential therapeutic target across leukemia subtypes.

#### 3.2.2 Mediating drug resistance

In leukemia, Gal-3 plays a multifaceted role in modulating drug resistance through stromal-leukemic crosstalk and intracellular signaling pathways (**Figure 3B**). In CML, primary cells overexpress Gal-3 along with activation of Akt/Erk/Mcl-1 pathways, conferring resistance to Bcr-Abl tyrosine kinase inhibitors and genotoxic agents by impairing apoptosis (38). AML-derived extracellular vesicles have been shown to stimulate mesenchymal stromal cells (MSCs) to upregulate Gal-3, which in turn protects leukemic cells against chemotherapy-induced cytotoxicity and contributes to disease relapse (43, 44). In AL, MSC-derived Gal-3 modulates the PI3K/Akt/GSK-3β axis, stabilizing β-catenin and activating Wnt/β-catenin signaling, thus promoting drug resistance (36). In pre-B ALL, stromal cell-derived Gal-3 induces an autocrine feedback loop that enhances its mRNA expression and sustains tonic activation of the NF-κB signaling pathway, establishing a chemoprotective microenvironment (45, 46). Furthermore, Gal-3 functions as a key mediator of crosstalk between BCP-ALL cells and the bone marrow stromal cells, thus promoting microenvironment-driven therapeutic resistance (47). Gal-3 emerges as a key molecular player in leukemia, critically driving pathological progression and therapeutic resistance across disease contexts.

Altogether, Gal-3 is a clinical biomarker and a molecular driver in leukemia. Its overexpression signifies poor prognosis and relapse risk, while therapeutic targeting of Gal-3 and associated pathways may reverse drug resistance and enhance chemotherapy efficacy. Future research should focus on the development of highly selective Gal-3 inhibitors and evaluate their therapeutic potential in combination with immunotherapeutic or targeted agents to overcome microenvironment-mediated treatment resistance and enhance long-term clinical outcomes.

## 4 Role of Gal-9 in leukemia

### 4.1 Gal-9 as a diagnostic and prognostic indicator in leukemia

Recent evidence highlights the crucial role of Gal-9 in leukemogenesis, disease progression, and clinical outcomes in AML and CLL. In AML, elevated Gal-9 expression is strongly associated with immune evasion mechanisms. It serves as a predictor of poor prognosis, particularly in post-hematopoietic stem cell transplantation (HSCT) relapse, where its upregulation correlates with adverse survival outcomes (48, 49). Gal-9 has emerged as a promising biomarker for assessing therapeutic effect, particularly in patients receiving azacytidine and venetoclax-based regimens (50). In CLL, serum Gal-9 levels are significantly elevated and show strong associations with advanced clinical stages, as defined by the Binet classification, and high-risk cytogenetic abnormalities, such as 17p deletion. Furthermore, elevated Gal-9 correlates with poor prognostic indicators, including shortened progression-free survival (PFS), reduced time to treatment (TTT), and resistance to therapy (51–55). Gal-9 shows high sensitivity and specificity in detecting disease progression and stratifying high-risk CLL subgroups (52). Persistent upregulation of Gal-9 after treatment predicts therapeutic failure, whereas lower post-treatment levels are typically observed in patients who attain remission or maintain stable disease (54). These findings position Gal-9 as a potent independent prognostic biomarker, with potential applications in monitoring disease progression, predicting therapeutic efficacy, and informing risk-adapted management strategies in both AML and CLL.

### 4.2 Molecular mechanisms underlying Gal-9-driven leukemogenesis

#### 4.2.1 Promoting leukemogenesis and immune evasion

**Figure 4:**
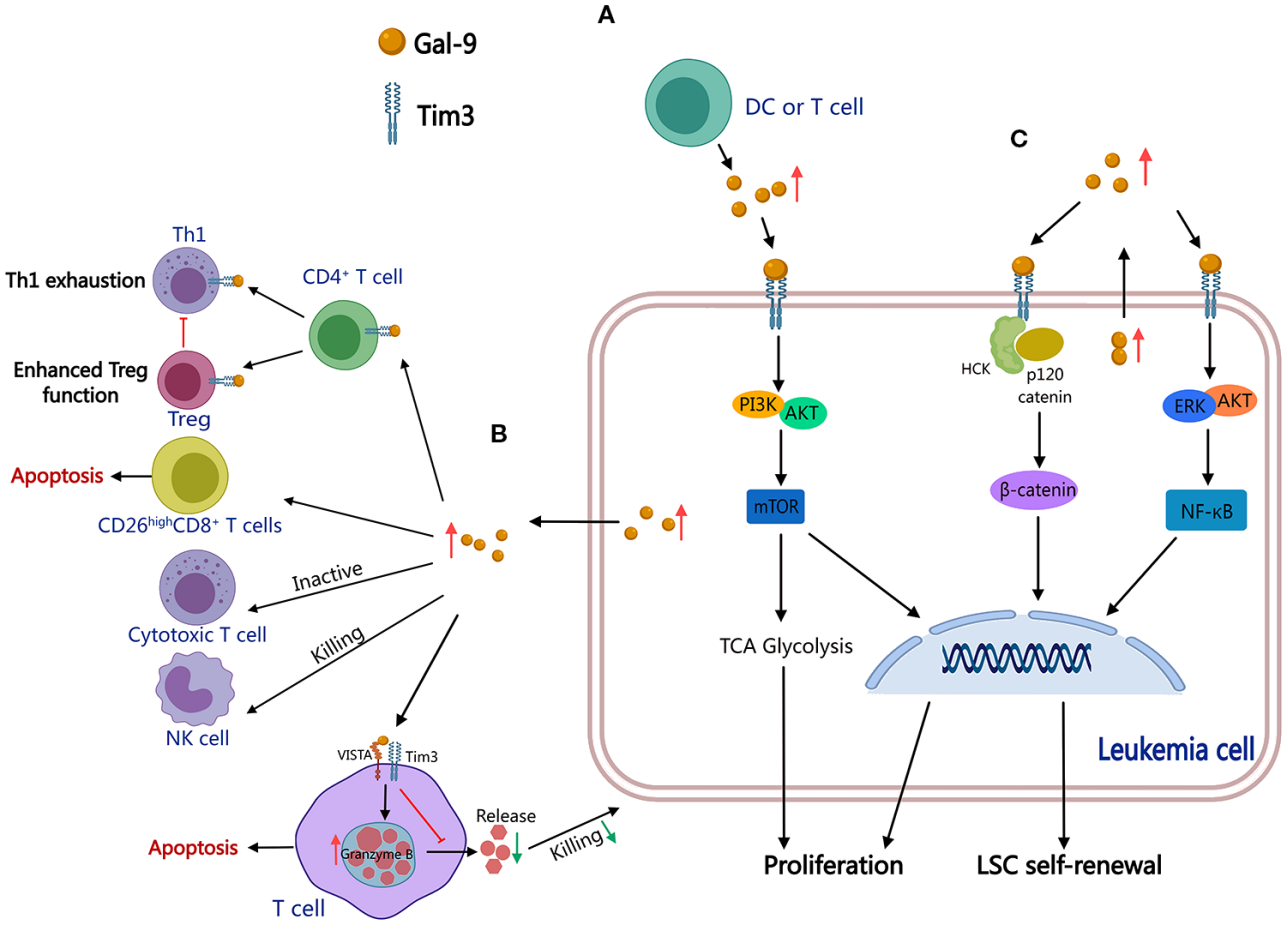
Molecular mechanisms associated with Gal-9-induced leukemogenesis. Gal-9 interacts with TIM-3 to activate the PI3K/Akt/mTOR pathway and promote proliferation **(A)**, impairing systemic T/NK cell function to suppress host anti-tumor immunity and promote immune evasion **(B)**, and co-activates the NF-κB/β-catenin pathways to promote self-renewal of LSC **(C)**. Thus, Gal-9 functions as a key mediator in multiple hematological malignancies. (This diagram was created using MedPeer).

Gal-9 is multidimensional in the pathobiology of leukemia, actively contributing to leukemogenesis and promoting immune escape mechanisms in AML and CLL. In AML, the Gal-9/TIM-3 interaction enhances leukemic cell survival by activating PI3K/Akt/mTOR and ERK pathways and upregulating glucose-6-phosphate dehydrogenase (G6PD) expression and glutathione levels, thus suppressing oxidative stress and facilitating proliferation (**Figure 4A**) (56, 57). Simultaneously, AML-derived Gal-9 binds to VISTA and TIM-3 receptors on T cells, forming an immunosuppressive complex that triggers granzyme B-mediated apoptosis and suppresses cytotoxic T lymphocyte (CTL) activity against leukemic cells (**Figure 4B**) (58). This Gal-9/TIM-3 axis further impairs anti-cancer functions of both CTLs and natural killer (NK) cells (**Figure 4B**) (49, 59). In CLL, Gal-9 contributes to immune escape by negatively regulating CD4^+^ T cells, suppressing Th1 effector responses, and promoting regulatory T cell (Treg) expansion (**Figure 4B**) (51). Gal-9 indirectly impairs anti-tumor immunity by depleting polyfunctional CD26^high^ CD8^+^ T cells, a subset characterized by enhanced migration potential, stem-like features, and proliferative potential (**Figure 4B**) (60). Gal-9 functions as a key mediator of leukemic cell survival, self-renewal, and immune evasion through dual mechanisms: directly enhancing the resilience of malignant cells and systemically suppressing host anti-tumor immunity by impairing T/NK cell function (61). These results highlight Gal-9 as a promising therapeutic target for restoring immune surveillance in leukemia.

#### 4.2.2 LSC self-renewal and therapy resistance

Gal-9 is pivotal in promoting LSC self-renewal and chemoresistance in multiple hematologic malignancies. Gal-9, *via* interaction with TIM-3, co-activates NF-κB and β-catenin signaling pathways, promoting LSC maintenance and driving disease progression in preleukemic and leukemic conditions, including myelodysplastic syndromes (MDSs), MPNs, CML, and AML (**Figure 4C**) (62–64). In AML, the Gal-9/TIM-3 axis further activates the hematopoietic cell kinase (HCK)/β-catenin signaling cascade, sustaining LSC propagation and self-renewal (**Figure 4C**) (65). Besides its involvement in stemness regulation, Gal-9 contributes to immunosuppression within the BM microenvironment, wherein MSCs exploit Gal-9 to reduce the cytotoxic efficacy of CAR-T cells, emphasizing its potential as a therapeutic target to prevent post-CAR-T relapse (66). Moreover, in B-ALL, adipocyte-induced Gal-9 expression has been shown to enhance chemoresistance, further highlighting its role in therapy resistance (67). These findings position Gal-9 as a key mediator of leukemic stemness, metabolic adaptation, and therapeutic resistance, advocating for targeted strategies to disrupt Gal-9-associated pathways in leukemia treatment.

Gal-9 is a key driver of leukemia progression and immune evasion, correlating with advanced disease stages, poor prognosis, and therapeutic resistance across leukemia subtypes. Its primary role in maintaining malignant cell survival and suppressing anti-tumor immunity underscores its potential as a therapeutic target for disrupting disease persistence and restoring treatment efficacy in leukemia.

## 5 Dual function of galectins

Growing evidence implicates Gal-1, Gal-3, and Gal-9 in the progression of leukemia through their roles in modulating TME interactions, preserving stemness characteristics, and facilitating the development of chemoresistance. Interestingly, emerging data also indicate that Gal-1 and Gal-9 may exert direct cytotoxic effects on leukemic cells under specific experimental conditions. The Gal-1 homolog RCG1 from *Rana catesbeiana* exerts potent growth inhibition in K562 (CML), HL-60 (APL), and U937 (histiocytic lymphoma) cells, with concurrent induction of substantial cellular aggregation, while human Gal-1 triggers apoptosis in Jurkat T-leukemia cells, an effect that was competitively inhibited by the specific galectin antagonist GB1490 (68, 69). Further investigations have elucidated the context-dependent functional duality of Gal-9 in monocytes, wherein intracellular localization promotes proinflammatory responses, while extracellular Gal-9 induces apoptosis, underscoring its compartment-specific activity (70). In AML, Gal-9 selectively targets both AraC-sensitive and AraC-resistant leukemic cell lines and primary CD34^+^ AML stem cells while showing synergistic cytotoxicity in combination with azacytidine. This selective activity spares healthy hematopoietic stem cells (71), highlighting its potential as a targeted therapeutic agent in AML. In CML, Gal-9 induces apoptosis in TKI-resistant Bcr-Abl+ cells through ATF-Noxa pathway activation, and in multiple myeloma, it activates the JNK/p38-H2AX axis to drive DNA damage responses (72, 73). In T-ALL models, Gal-9 suppresses cell proliferation and clonogenicity by modulating Bax/Bcl-2 ratios and activating caspase-3-dependent apoptosis (74). These results position Gal-1 and Gal-9 as multifaceted therapeutic candidates capable of targeting leukemic cells across various disease stages and resistance profiles, with their mechanisms of action controlled by cellular context and specific signaling pathway activation.

## 6 Gal-1, -3, and -9 targeting strategies in leukemia

Although Gal-1, -3, and -9 play significant roles in leukemia pathogenesis, progression, invasion, and stemness maintenance, their functional outcomes exhibit starkly contrasting effects contingent upon cellular states and microenvironmental contexts. Therefore, developing highly specific inhibitors represents a critical imperative (2). Recent advancements in understanding the pathophysiological functions of galectins have catalyzed the development of diverse therapeutic agents targeting Gal-1, -3, and -9, some of which have been evaluated in clinical trials (**Table 1**). Available pharmacological strategies can be classified into four categories (1): small-molecule inhibitors targeting the CRDs of galectins; (2) modified polysaccharides and their synthetic analogs designed to interfere with galectin–glycan interactions; (3) peptide-based inhibitors and peptidomimetics with high binding specificity; and (4) biologic therapeutics, including siRNA-based platforms, high-affinity aptamers, engineered truncated galectin variants, and monoclonal neutralizing antibodies. Despite significant advances in targeted therapeutic development, progress in translating galectin inhibitors into clinical applications for leukemia treatment remains limited. To date, only a limited number of Gal-1, -3 and -9 inhibitors have advanced to preclinical or clinical evaluation specifically focused on leukemia.

**Table 1:** Completed and current clinical trials of Gal-1, -3, and -9 agonists.

Targets	Agents	Diseases	Trail number	Phase	Trial Status
Gal-1	OTX008	Advanced Solid Tumors (75, 76)	NCT01724320	Phase 1	Unknown
Gal-1 and Gal-3	GM-CT-01	Solid Tumors (77)	NCT00054977	Phase I	Completed
		Biliary Cancer	NCT00386516	Phase 2	Withdrawn
		Colorectal Cancer	NCT00388700	Phase 2	Withdrawn
Gal-3	GR-MD-02	Melanoma (78)	NCT02117362	Phase 1	Completed
		Non-small Cell Lung Cancer (78)	NCT02575404	Phase 1	Completed
		Squamous Cell Head and Neck Cance (78)			
		Non-Alcoholic Steatohepatitis (NASH) (79, 80)	NCT02421094	Phase 2	Completed
		NASH Cirrhosis (81)	NCT04365868	Phase 2b/3	Active
		Psoriasis (82)	NCT02407041	Phase 2	Completed
	GB1211	Non-Small Cell Lung Cancer (83, 84)	NCT05240131	Phase 2	Active
	GB0139/ TD139	Idiopathic Pulmonary Fibrosis (85, 86)	NCT03832946	Phase 2b	Completed
	MCP	Hypertension (87, 88)	NCT01960946	Not Applicable	Completed
		Prostate Cancer (89, 90)	NCT01681823	Phase 2	Completed
		Osteoarthritis (91, 92)	NCT02800629	Phase 3	Unknown
	GCS-100	Multiple Myeloma (93, 94)	NCT00609817	Phase 1	Terminated
		Chronic Kidney Disease	NCT02155673	Phase 2	Completed
		**Chronic Lymphocytic Leukemia** (95)	**NCT00514696**	**Phase 2**	**Completed**
	TB006	Autism Spectrum Disorder (96, 97)	NCT06500637	Phase 2	Recruiting
	ProLectin M	COVID-19 (98)	NCT05733780	Phase 2	Active
Gal-9	LYT-200	**Acute Myeloid Leukemia** (99)	**NCT05829226**	**Phase 1**	**Recruiting**
		Metastatic Solid Tumors (100)	NCT04666688	Phase 2	Completed

Bold values denote clinical trials for leukemia. (Data obtained from www.clinicaltrials.gov).

**Figure 5:**
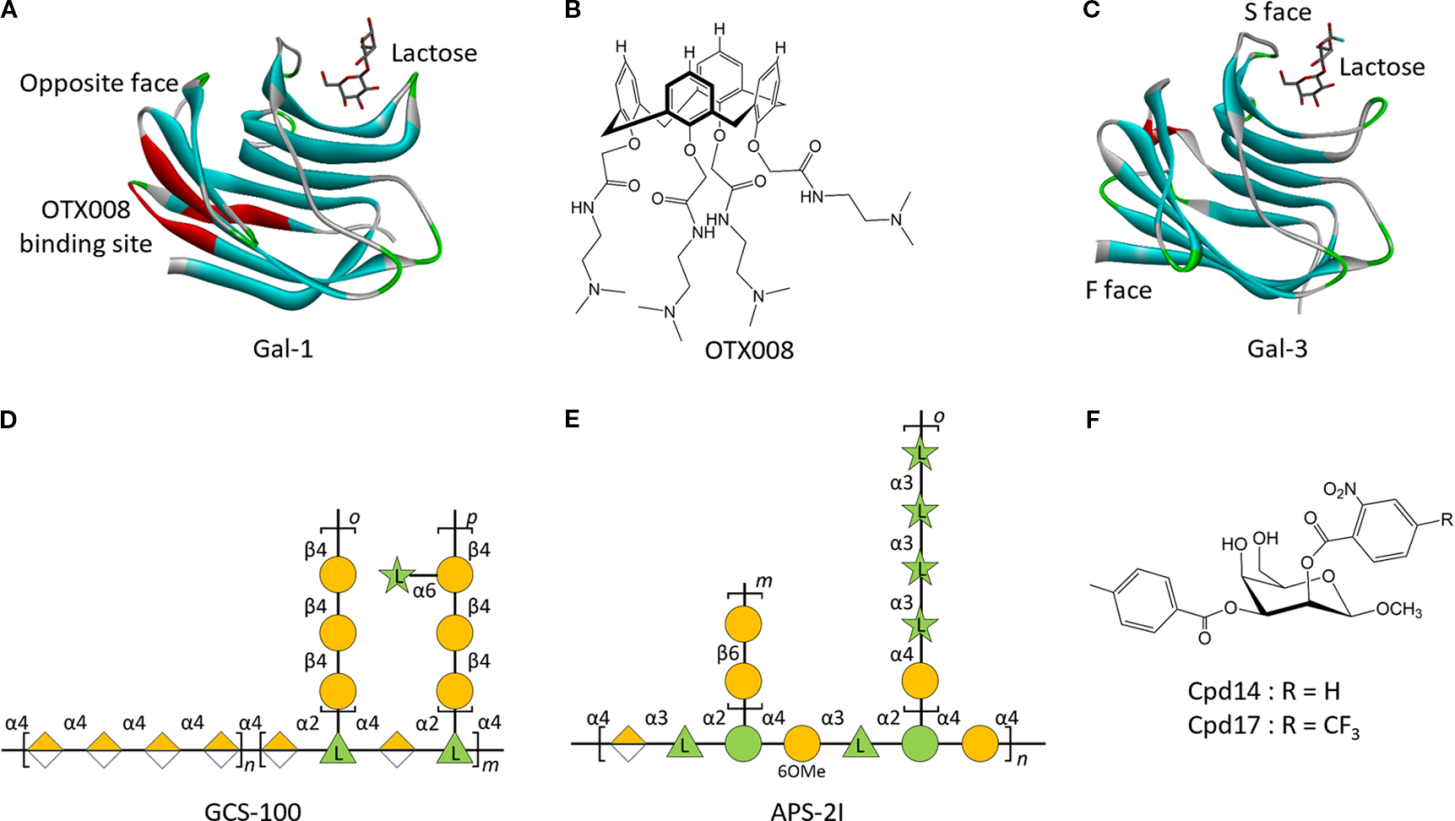
Structure of galectins and their inhibitors in leukemia. **(A)**, Crystal structure of human Gal-1 CRD in complex with Lactose (PDB 1W6O), and its binding site for OTX008. **(B)**, Structures of OTX008. **(C)**, Crystal structure of human Gal-3 CRD in complex with Lactose (PDB 3ZSJ). Structures of modified citrus pectin (MCP) polysaccharides GCS-100 **(D)**, APS-2I **(E)**, and Cpd14/17 **(F)**. *n*, *m*, *o*, and *p* represent the number of repetitive residues in the polysaccharide.

Gal-1 is a 14-kDa protein comprising 135 amino acids encoded by the *LGALS1* gene. It is a non-covalently stabilized homodimer, adopting a sandwich-like conformation formed by two antiparallel β-sheets, each containing a galactoside-binding site in the CRD (**Figure 5A**) (101). Pharmacological inhibition of Gal-1 has shown anti-leukemic efficacy through dual targeting of both malignant cells and the supportive TME. OTX008, a non-peptidic calixarene-based Anginex mimetic (**Figure 5B**), binds to the noncanonical face of Gal-1’s CRD (**Figure 5A**), thus disrupting lactose binding and attenuating Gal-1-mediated biological functions (102). In BCP-ALL, OTX008 inhibits leukemic cell proliferation, migration, and adhesion, while enhancing chemosensitivity, thus exerting therapeutic effects on the leukemic clone and its protective niche (103). OTX008 has also been shown to overcome ibrutinib resistance in CLL, underscoring its potential as a promising therapeutic agent in drug-resistant hematologic malignancies (104).

Gal-3, a β-galactoside-binding lectin, features three distinct structural domains: an N-terminal domain, a collagen-like sequence, and a C-terminal CRD (**Figure 5C**). The CRD contains two functional grooves, the canonical S-face that recognizes β-galactosides (e.g., lactose) and the non-canonical F-face that interacts with complex glycans (e.g., GM1/MCPs) (105). Allosteric modulation induced by ligand binding at one interface of the CRD results in a reciprocal reduction in binding affinity at the opposing site, thus illustrating a competitive interplay between the two recognition domains (106, 107). This structural bifunctionality has provided significant understanding for the rational design of isoform-specific therapeutic agents targeting galectins. The Gal-3 inhibitor GCS-100 (**Figure 5D**) demonstrates anti-leukemic activity across multiple AML cell lines (OCI-AML3, THP-1, HL60) through dual mechanisms: cell cycle arrest *via* Cyclin E/D2 downregulation and caspase-8/-9-dependent apoptosis induction (95, 108). GCS-100 synergizes with BH3 mimetics to enhance multi-pathway apoptotic signaling, amplifying its cytotoxic efficacy (109). Clinical evaluation in a phase II trial for chronic lymphocytic leukemia (NCT00514696) has validated its therapeutic potential, with 50% patients achieving stable disease and 25% exhibiting a partial response (including >50% shrinkage of lymph node lesions), thereby supporting further translational development (110). In addition to synthetic inhibitors, naturally derived compounds have shown selective Gal-3-modulating activity in leukemia. Olive pectin extracts have been shown to selectively activate Gal-3-dependent caspase-3 signaling in AML cells (111). Similarly, Angelica *sinensis* polysaccharide APS-2I (**Figure 5E**) antagonizes the anti-apoptotic function of Gal-3 by reactivating caspase-3 signaling, resulting in significantly prolonged survival in murine models (112). Complementing these macromolecular approaches, synthetic carbohydrate-based inhibitors Cpd14 and Cpd17 (**Figure 5F**), derived from taloside scaffolds, show a synergistic cytostatic and cytotoxic effect in BCP-ALL. These agents impair leukemic cell adhesion and migration, thus reducing metastatic potential, while inducing apoptotic cell death (113, 114). These findings provide multifaceted strategies for Gal-3-targeted therapies.

Gal-9, encoded by the *LGALS9* gene on chromosome 17, features a characteristic sequence with a highly conserved CRD of approximately 130 amino acids (115, 116). As a tandem-repeat-type galectin, human Gal-9 exists in three isoforms—Gal-9(S), Gal-9(M), and Gal-9(L)—which differ in the length of the interdomain linker that separates the N- and C- CRDs (117). The structure and length of this linker region influence the formation of multivalent lattices, thereby modulating the protein’s capacity to bind glycan ligands. Although all isoforms exhibit potent activity, their expression patterns are not uniform. Differences in biological function have been reported: Gal-9(L) inhibits endothelial adhesion of colon cancer cells, whereas Gal-9(S) and Gal-9(M) promote it (118). In AML models, LYT-200 (an anti-galectin-9 monoclonal antibody) exerts direct anti-leukemic effects through inducing DNA damage and apoptosis. When combined with venetoclax and standard chemotherapy, LYT-200 prolongs survival and protects against long-term relapse *in vivo*. A Phase I clinical trial in the USA is currently evaluating the efficacy of LYT-200 monotherapy and combination therapy with VEN/hypomethylating agents (HMA) in patients with relapsed/refractory (R/R) AML or high-risk MDSs (NCT05829226) (99). In the LYT-200 monotherapy group (7.5 mg/kg), the clinical benefit rate, defined as the proportion of patients achieving stable disease (SD), partial response (PR), complete response (CR), CR with incomplete hematologic recovery (CRi), or morphologic leukemia-free state (MLFS), was 100%, with a partial response rate of 25% (99).

## 7 Challenge of Gal-1, -3, and -9 inhibitors

The development of galectin-targeted inhibitors is impeded by modality-specific and broadly shared challenges, necessitating integrated, interdisciplinary strategies. Although Gal-1, Gal-3, and Gal-9 show overlapping roles in leukemic progression, their distinct pathological functions in diverse disease contexts necessitate therapeutics with a high degree of isoform specificity. A basic challenge arises from the substantial structural homology (ranging from 20% to 50%) shared among the CRDs of galectin family members. These domains engage β-D-galactopyranoside-containing glycans through highly conserved binding mechanisms, thus complicating the selective design of inhibitors targeting individual isoforms (119). This molecular mimicry increases risks of off-target interactions with both non-target galectins and structurally analogous cellular proteins, potentially negating therapeutic efficacy or inducing paradoxical effects. Moreover, the development of galectin inhibitors is complicated by the need to balance dual requirements of potency and selectivity, as insufficient specificity may lead to off-target effects that compromise the physiological roles of galectins in immune modulation and tissue homeostasis.

**Figure 6:**
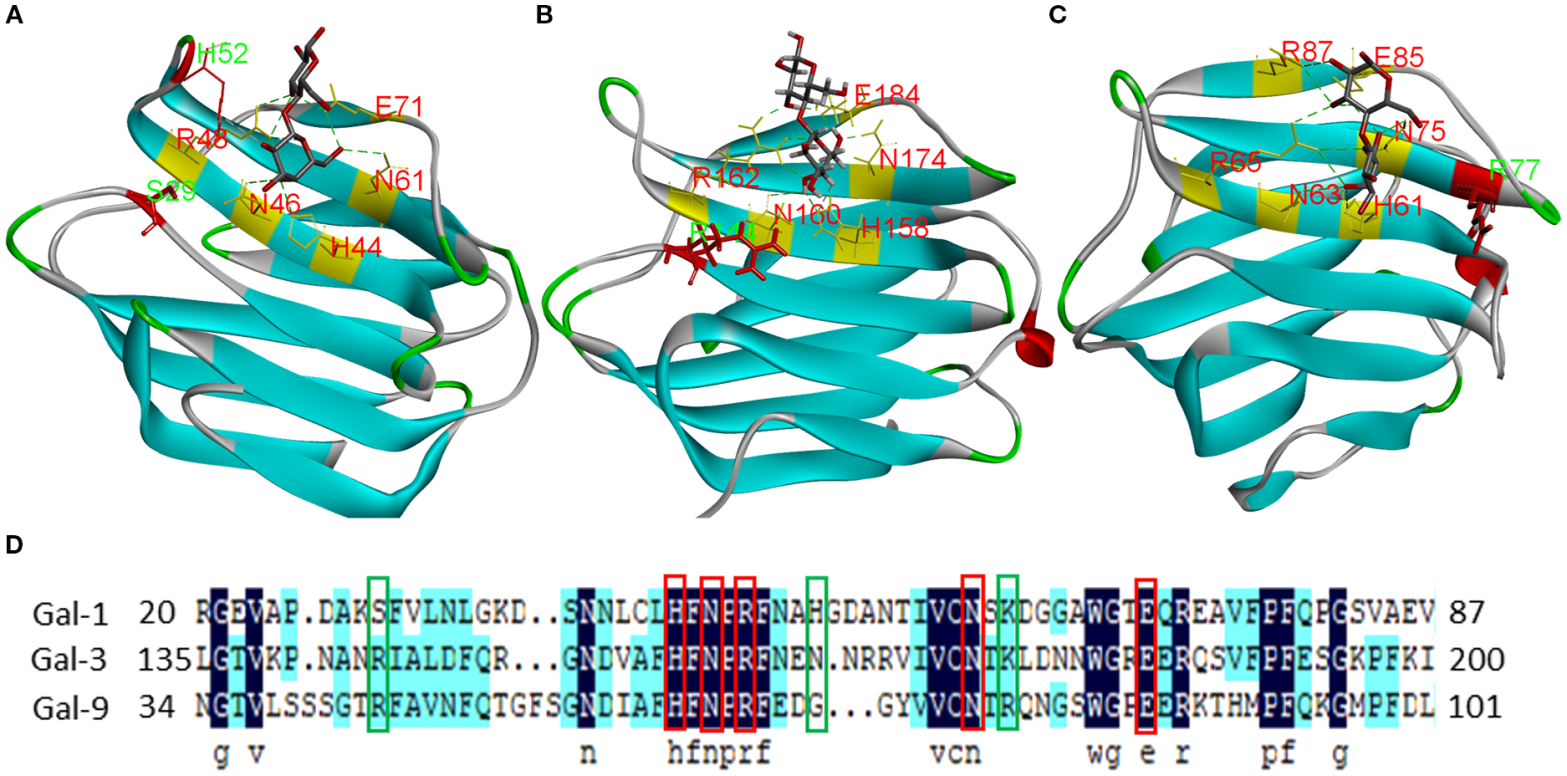
Structures and ligand-binding sites of Gal-1, -3, and -9. Crystal structures of the CRDs of human **(A)** Gal-1 (PDB: [2ZKN]), **(B)** Gal-3 (PDB: [6EYM]), and **(C)** Gal-9 (PDB: [2EAK]) in complex with lactose. Conserved lactose-binding site residues are highlighted in red; residues conferring unique structural features are shown in green. **(D)** Alignment of Gal-1, -3, and -9 sequences.

The development of selective galectin inhibitors requires a comprehensive understanding of both conserved binding motifs and subtype-specific structural features. Gal-1, -3, and -9 share a conserved subsite architecture mediated by key amino acids (Gal-1: H44, N46, R48, N61, E71 (**Figure 6A**); Gal-3: H158, N160, R162, N174, E184 (**Figure 6B**); Gal-9: H61, N63, R65, N75, E85 (**Figure 6C**)) that mediate hydrogen-bond interactions with inhibitors (8, 120, 121). However, the high structural homology among galectin family members complicates the design of subtype-selective compounds. To overcome this challenge, rational drug design should focus on the strategic exploitation of unique residue features within the binding pockets of individual galectin isoforms, namely, Ser29 and His52 in Gal-1, Arg144 in Gal-3, and Arg77 in Gal-9 (**Figure 6D**, green boxes) (8, 121, 122). These structurally divergent residues are critical pharmacophoric determinants for engineering selective affinity. By optimizing interactions with these isoform-specific residues while preserving affinity for conserved carbohydrate recognition motifs, it may be possible to develop inhibitors with improved target specificity and minimized off-target effects.

In addition to these barriers, modality-specific challenges further complicate galectin-targeted drug development. Small-molecule galactoside and lactoside analogs demand complex synthetic strategies and optimization, often surpassing the production complexities encountered with biologic therapeutics. To address these multifaceted challenges, the integration of structural glycomics, CRD engineering, and pharmacokinetic modeling is essential. This multidisciplinary approach is crucial for the rational design and advancement of clinically effective galectin-targeted therapeutics within this intricate and evolving therapeutic landscape.

## 8 Conclusion

In conclusion, Gal-1, -3, and -9 have emerged as key regulators of leukemia pathogenesis, exerting multifaceted effects on leukemic cell survival, therapeutic resistance, and immune evasion. Their overexpression is consistently associated with poor clinical outcomes, underscoring their potential as prognostic biomarkers and therapeutic targets. Many preclinical evidence, along with ongoing clinical studies, supports the feasibility of galectin-directed therapies as a novel approach to overcoming current treatment limitations. As challenges, i.e., drug resistance and disease relapse, continue to impede effective leukemia management, the development of galectin inhibitors holds promise for advancing precision medicine. Further research is needed to elucidate the context-dependent roles of individual galectins and optimize targeted clinical translation strategies.
